# Marital status and survival in cancer patients: A systematic review and meta‐analysis

**DOI:** 10.1002/cam4.5003

**Published:** 2022-07-04

**Authors:** Kaja Krajc, Špela Miroševič, Jakob Sajovic, Zalika Klemenc Ketiš, David Spiegel, Gorazd Drevenšek, Martina Drevenšek

**Affiliations:** ^1^ Faculty of Mathematics, Natural Sciences and Information Technologies University of Primorska Koper Slovenia; ^2^ Department of Family Medicine, Faculty of Medicine University of Ljubljana Ljubljana Slovenia; ^3^ Department of Stomatology University Medical Centre Ljubljana Ljubljana Slovenia; ^4^ Department of Family Medicine, Faculty of Medicine University of Maribor Maribor Slovenia; ^5^ Community Health Centre Ljubljana Ljubljana Slovenia; ^6^ Department of Psychiatry and Behavioural Sciences Stanford University School of Medicine Stanford California USA; ^7^ Institute of Pharmacology and Experimental Toxicology, Faculty of Medicine Ljubljana University of Ljubljana Ljubljana Slovenia

**Keywords:** marital status, neoplasms, social support, survival, systematic review

## Abstract

**Background:**

In recent years, authors have repeatedly reported on the significance of social support in cancer survival. Although overall the studies appear to be convincing, little is known about which types of social support promote better survival rates, and which subgroups of cancer patients are more susceptible to the benefits of it. The aim of this study was to identify, organize, and examine studies reporting on the significance of social support in cancer survival.

**Methods:**

The PubMed, CINAHL and EBSCO databases were searched using the keywords social support/marital status, cancer, and survival/mortality. Where possible we used a meta‐analytical approach, specifically a random effect model, in order to combine the results of the hazard ratios in studies from which this information could be obtained. When interpreting clinical relevance, we used the number needed to treat (NNT).

**Results:**

Better survival was observed in married patients when compared to unmarried (single, never‐married, divorced/separated, and widowed) in overall and cancer‐specific survival. Gender group differences showed that the association was statistically significant only in cancer‐specific survival when comparing divorced/separated male and female cancer patients (*p* < 0.001), thus confirming results from the previous meta‐analysis.

**Conclusions:**

Being unmarried is associated with significantly worse overall and cancer‐specific survival. The most vulnerable group found in our study were divorced/separated men. The results of this review can motivate physicians, oncologists, and other healthcare professionals to be aware of the importance of patients' social support, especially in the identified sub‐group.

## Graphical abstract

Being married is associated with significantly better overall survival. Being unmarried male is associated with significantly worse survival. Marital status should be taken into consideration when providing cancer care.

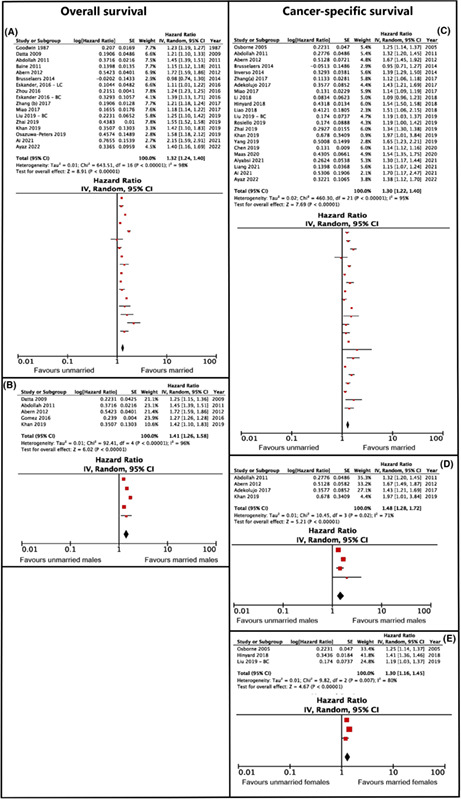

## 1 INTRODUCTION

Cancer is a disease with a global health burden; it is a leading cause of deaths worldwide.^1^ While several important risk factors have been identified (e.g. tobacco use, cancer‐causing infections, high body mass index, etc.), data on how psychosocial factors impact on cancer survival is less evident. Researchers have been exploring the association between social support and cancer survival since 1980 in many naturalistic (non‐interventional) and interventional studies. A recent meta‐analysis exploring the effect of randomized‐controlled trials on cancer survival concluded that the overall effect favors groups that receive psychosocial treatment. Interestingly, the effect was higher in studies that included more unmarried patients.^2^

Unmarried cancer patients are more likely to be diagnosed with an advanced stage of the disease than married patients,^3^, ^4^, ^5^ who often have a higher socioeconomic status than unmarried ones, enabling them to have better access to healthcare.^6^ They can also receive instrumental support from their spouse (e.g. assistance with transportation, paperwork, household chores) so they can fully focus on their treatment. Importantly, a partner can provide emotional support, which can mitigate the stress of cancer treatment.^7^

Marital status has been found to be an independent predictive factor associated with better odds of survival in various cancer types.^8^, ^9^ The effect of the social support provided by a partner may be physiologically mediated through neuroendocrine, nervous, and immune interactions which are directly related to cancer.^10^ For example, cancer patients who have a higher quality of social support have greater activity in natural killer (NK) cells, which are important cytotoxic cells of the immune system and can recognize and destroy cancer cells.^10^ The hormone oxytocin, which is released during social interactions, may also indirectly inhibit the growth of cancer cells by inhibiting the stress response.^11^

To date, two systematic reviews and meta‐analyses exist on the topic of social support and cancer survival.^12^, ^13^ They report a 12% decrease mortality risk in married patients and that never‐married patients had a worse survival rate than widowed or divorced/separated patients.^12^ Furthermore, divorced/separated men had a 12% higher risk of cancer mortality compared to the 9% mortality rate in women, thus drawing attention to the importance of the role of gender.^13^ However, the study only evaluated the association between marital status and survival by gender, excluding a general marital status—cancer survival analysis. Importantly, their literature search was carried out in 2018, and this area of research experienced significant growth after their publications.

The aim of this paper is to examine the association between marital status and different groups of unmarried cancer patients (e.g. divorced/separated, single, widowed) on overall and cancer‐specific survival. As the different groups of unmarried patients likely experience different levels of stress, the examination of survival by group can unveil new data on the link between marital status and cancer survival. We set the following objectives for the review and meta‐analysis:

- To analyze the difference between overall and cancer‐specific survival according to marital status (i.e., married, unmarried, never‐married single, divorced/separated and widowed)
- To examine which subgroup of cancer patients (e.g. gender, cancer stage) are associated with better overall and cancer‐specific survival.

## 2 METHODS

### 2.1 Literature search strategy

The study followed the PRISMA statement (Preferred Reporting Items for Systematic Review and Meta‐Analysis). Relevant articles were identified through the PubMed, CINAHL, and EBSCO databases between January and June 2018, and regularly updated up to April 2022 (see Table S1 for the specified search strategy for EBSCO and PubMed). Additional articles were obtained by searching through the reference lists of the included studies.

### 2.2 Inclusion and exclusion criteria

The review included eligible studies that: (1) were published as original articles; (2) analyzed adult cancer patients (>18 years); (3) reported a correlation between marital status and survival, such as overall survival (OS) or cancer‐specific survival (CSS); (4) provided clear data from which to directly extract hazard ratios (HR) and 95% confidence intervals (CI). Articles were excluded if: (1) they analyzed patients with childhood cancer; (2) the paper was a republished report; (3) the effect of marital status on cancer survival was not a primary outcome; (4) and they were not published in English.

### 2.3 Data collection and quality assessment

Three authors independently examined all the selected publications and extracted data in accordance with the following protocol. From each of the selected articles, we obtained the following data: first author; year of publication; country; type of longitudinal study (prospective/retrospective); sample size (*n*); recruitment; follow‐up time expressed in years; sex (the percentage of male and female subjects); age of participants (M, SD); diagnosis (type and stage of cancer); dependent variable (marital status); factors that were included in the adjusted analysis; and the conclusion concerning the association between marital status and survival (reported with hazard ratio (HR) and 95% cluster interval (CI)). Data were collated in a spreadsheet with columns denoting extracted data categories and rows denoting studies (see Table 1).

**TABLE 1:** Main characteristics of selected studies (*n* = 67)

			Study's characteristics				Patients' demographic characteristics		Patients' clinical characteristics			
First author	Year	Country	Type of study	Sample size	Recruitment	Follow‐up (years)	Sex (%)	Age (M, SD)	Type of cancer	Stage of cancer	Dependent variable	Adjusted analysis
Goodwin^23^	1987	New Mexico	Retrospective	27,706	Tumor Registry	5	ND	ND	Various	I–IV	Marital status	Stage at diagnosis and definitive treatment.
Osborne^39^	2005	USA	Retrospective	32,268	SEER	3	Women	ND	Breast cancer	I–IV	Marital status	Age, ethnicity, SEER area, tumor size, stage, grade, estrogen receptor status, comorbidity index score, treatment variables, chemotherapy, census tract education level and census tract household income in quartiles.
Reyes Ortiz^57^	2007	USA	Retrospective	14,630	SEER	5	W (39.9), M (60.1),	75.2 (6.9)	Melanoma	I–IV	Marital status	Age, gender, race/ethnicity, socioeconomic status, histology, site, stage at diagnosis and comorbidity.
Saito‐Nakaya^63^	2008	Japan	Prospective	1230	Thoracic Oncology Division, National Cancer Center Hospital East	5	W (29.7), M (70.3),	ND	Non‐small cell lung cancer	I–IV	Marital status	age, BMI, education, PS, histology type, smoke stage, definitive treatment, and HADS‐depression adjusted.
Datta^28^	2009	USA	Retrospective	19,982	SEER	5	W (25.8), M (74.2)	60–80+ (ND)	Bladder cancer	II–IV	Marital status	Age, race, number of comorbidities, receipt of treatment, socioeconomic status, and teaching hospital designation, stage at diagnosis.
Patel^52^	2010	USA	Retrospective	7997	SEER	5	Women	ND	Cervical cancer	I–IV	Marital status	
Abdollah^26^	2011	USA	Retrospective	163,697	SEER	ND	Men	63	Prostate cancer	I–IV	Marital status	Age, race, socioeconomic status, tumor grade, tumor stage, lymph node stage, year of surgery
Baine^31^	2011	USA	Retrospective	34,555	SEER	ND	W (48.6), M (51.4)	69	Pancreatic cancer	I–IV	Marital status	Gender, race, age at diagnosis, year of diagnosis, cancer‐directed surgery, radiation therapy and stage.
Wang^86^	2011	USA	Retrospective	127,753	SEER	5	W (52.5), M (47.5)	ND	Colon cancer	I–IV	Marital status	Age, cancer stage, race and surgery receipt.
Abern^30^	2012	USA	Retrospective	20,245	SEER	10	Men	35.4	Testis cancer	I–II	Marital status	Age, stage at diagnosis, Race, Histologic type, Year of diagnosis, Region
Tannenbaum^78^	2013	USA	Retrospective	161,228	The Florida Cancer Data System and Florida's Agency for Health Care Administration	5	W (44.3), M (55.7)	69.8 (11.2)	Lung cancer	I–IV	Marital status	Race/Ethnicity/SES, demographics + clinical + individual comorbidities.
Aizer^85^	2013	USA	Retrospective	734,899	SEER	3.1	W (48.1), M (51.9)	64.5 (13)	Various	I–IV	Marital status	Age, gender, race, income, education, residence type, stage, primary site and type of treatment.
Mahdi^66^	2011	USA	Retrospective	49,777	SEER	2.2	Women	ND	Epithelial ovarian cancer	I–IV	Marital status	Age, race, histology, stage, grade, lymphadenectomy and extent of surgery.
Brusselaers^22^	2015	Sweden	Prospective	606	Swedish Hospitals	5	M (80.4), W (19.6)	23.9% <60, 76.1% > 60	Esophageal cancer	I–IV	Marital status	Sex, age, tumor stage, histology, major complications, comorbidity and surgeon volume.
Inverso^41^	2014	USA	Retrospective	51,272	SEER	1.6	W (25.1), M (74.9),	61 (12.9)	Head and neck cancer	I–IV	Marital status	Age at diagnosis, gender, race, income, level of education, residence type and definitive treatment.
Li^67^	2015	USA	Retrospective	112,776	SEER	5	W (48.6), M (51.4)	ND	Colorectal cancer	I–IV	Marital status	Age, race, grade, histotype and TNM stage.
Wang^65^	2016	USA	Retrospective	13,370	SEER	1.1	W (49.43). M (50.57)	32.8% ≤60, 67.2% >60	Pancreatic cancer	I–IV	Marital status	Primary site location, age, race, year of diagnosis, tumor size (cm), SEER stage
Zhou^36^	2016	USA	Retrospective	18,815	SEER	5	W (37.6), M (62.4)	ND	Gastric cancers	I–IV	Marital status	Age, race, pathological differentiation, histological type, TNM stage, surgery and radiotherapy.
Eskander^24^	2016	USA	Retrospective	11,849	Tumor registry	1	W (63.8), M (36.2)	ND	Various	I–IV	Marital status	Gender, age, insurance status, race; lung: surgery models, type of therapy and stage.
Shi^58^	2016	USA	Retrospective	61,077	SEER	19	W (78.1), M (21.9)	47.7 (14.8)	Thyroid cancer	I–IV	Marital status	Gender, age, race, follicular vs. papillary, T3/4 vs. T1/2, N stage, distant metastasis, surgery procedure, lobectomy and adjuvant therapy.
Jin^68^	2016	USA	Retrospective	18,196	SEER	M = 2 (1–100)	W (36.7), M (63.3)	ND	Gastric cancer	I–IV	Marital status	Age, gender, race, tumor location, histological type, differentiated grade, stage, and year of diagnosis.
He^69^	2017	USA	Retrospective	40,809	SEER	ND	W (25.4), M (74.6)	47.1% <60, 52.9% >60	Liver cancer	I–IV	Marital status	Gender, age, race, grade, hystotype, SEER stage, type of therapy
Adekolujo^40^	2016	USA	Retrospective	3761	SEER	5	Men	Married 64.8, Unmarried 65	Breast cancer	I–IV	Marital status	Age, race, median household income, stage, grade, combined ER/PR status, histological type, and surgical treatment
Du^53^	2017	USA	Retrospective	69,139	SEER	M = 1.3	W (24.0), M (76.0)	67 (11.7)	Esophageal cancer	I–IV	Marital status	Age, gender, race/ethnicity, household income, histology, tumor site, SEER stages, therapy, and insurance status
Zhang (a)^62^	2017	USA	Retrospective	16,910	SEER	5	W (44.0), M (56.0)	26.0% <57, 74.0% >57	Gastric cancer	I–IV	Marital status	Site, sex, race, age, grade, histotype, TNM stage, surgery type and selection of radiotherapy
Miao^34^	2017	USA	Retrospective	112,860	SEER	5	W (36.6), M (63.4),	38.7% <60, 61.3% >60	Kidney cancer	I–IV	Marital status	Sex, age, race, grade, TNM, SEER stage, type of therapy
Li^60^	2017	USA	Retrospective	6627	SEER	5	W (70.4), M (29.6)	24.9% <60, 75.1% >60	Gallbladder cancer	I–IV	Marital status	Age, race, grade, histologic type, AJJC stage, SEER
Rubin^51^	2017	USA	Retrospective	65	Boston University Medical Center	3	W (18.5), M (81.5)	61.58 (8.94)	Human papilloma virus‐positive oropharyngeal cancer	I–IV	Marital status	age, sex, race, insurance type, smoking status, treatment, and AJCC combined pathologic stage
Wang^71^	2017	USA	Retrospective	62,405	SEER	5	W (38.1), M (61.9)	46.5% <60, 53.5% >60	Renal cancer	I–IV	Marital status	Sex, age, race, tumor size, laterality, SEER stage, grade
Hinyard^21^	2017	USA	Retrospective	166,701	SEER	ND	Women	64.5 (24.1)	Breast cancer	I–IV	Marital status	Unadjusted analysis
Zhang (b)^35^	2017	USA	Retrospective	15,598	SEER	ND	W (19.3), M (80.7)	20.2% <55, 79.8% >56	Esophageal cancer	I–IV	Marital status	Sex, race, age, histology, grade, location, TNM stage, therapy
Wu^79^	2017	USA	Retrospective	70,006	SEER	M = 1.3	W (47.0), M (53.0)	23.3% <60, 76.7% >60	Non‐small cell lung cancer	ND	Marital status	Sex, age, race, diagnosis year, median household income, grade, TNM stage, histology, surgery, radiotherapy, radiotherapy
Alvi^87^	2018	USA	Retrospective	1188	SEER	10	W (57.5) M (42.5)	20+ (ND)	Spinal cord tumors	ND	Marital status	Age, gender, SES, insurance status
Chen^88^	2018	USA	Retrospective	6582	SEER	5	W (49.0) M (51.0)	18–70+ (ND	Gastrointestinal stromal tumor	Localized, regional, distant stage	Marital status	Sex, race, age histology, stage, surgery, radiotherapy
Liao^44^	2018	China	Retrospective	457	Cancer registry dataset of the Kaohsiung veteran's general hospital	5	W (7.7), M (92.3)	ND	Oral cavity cancer	I–IV	Marital status	T‐category, N Category, differentiation, neck dissection, adjuvant therapy
Niu^72^	2018	USA	Retrospective	133,846	SEER	5	W (24.2), M (75.8)	78.5% >60+ 21.5% <60	Bladder urothelial carcinoma	I–IV	Marital status	Sex, age, race, primary site, pathological grading, TNM stage, surgery
Wu^61^	2018	USA	Retrospective	4001	SEER	8	Women	Median 66	Vulvar cancer	I–IV	Marital status	Age, race, grade, tumor stage, nodal stage, M stage, surgery, radiotherapy, chemotherapy
Xie—a^73^	2018	USA	Retrospective	43,324	SEER	ND, ~10	W (42.6) M (57.4)	ND	Astrocytoma	I–IV	Marital status	Age, sex, race, WHO grade, diagnosis year, median household income, surgery
Xie—b^74^	2018	USA	Retrospective	30,767	SEER	ND, ~10	W (42.0) M (58.0)	ND	Glioblastoma multiforme	I–IV	Marital status	Sex, age, race, registry site, diagnosis year, education, median household income, insurance, laterality of cancer, surgery, metastasis, tumor size, SEER stage
Zhang^56^	2018	USA	Retrospective	18,013	SEER	5	W (49.7) M (50.3)	55.6% <60, 44.4% >60	Soft tissue sarcoma	I–IV	Marital status	Sex, age, race, diagnosis year, pathological grade, tumor size, SEER historic stage, insurance status, surgery
Li^38^	2018	USA	Retrospective	5196	SEER	5	W (30.7) M (69.3)	65+	Rectal cancer	I–IV	Marital status	Sex, age, year of diagnosis, race, stage, grade, chemotherapy, radiotherapy, and surgery type
Wang^81^	2018	USA	Retrospective	27,498	SEER	5	W (40.4) M (59.6)	40.8% <60, 59.2% ≥60	Rectal cancer	I–IV	Marital status	Sex, age, race, pathologic grade, histotype, adenocarcinoma, surgery, TNM stage
Liu^70^	2019	USA	Retrospective	824,554	SEER	5	ND	68.6 (9.05)	Prostate cancer	ND	Marital status	Age, race, Gleason score, surgery
Chen^46^	2019	USA	Retrospective	72, 984	SEER	10	W (54.2) M (45.8)	18–75+ (ND)	Non‐small cell lung cancer	I–IV	Marital status	Sex, race, age, histology, tumor stage, surgery, radiotherapy
Dong^82^	2019	USA	Retrospective	39,387	SEER	5	Women	Age range 18–80+, most 50–69 (ND)	Endometrial cancer	I–IV	Marital status	Age, diagnosis year, race, histology, grade of cancer (I–IV)
Liu^33^	2019	USA	Retrospective	1342	SEER	5	Women	51.6% =56+; 48.4% <56	Breast cancer	I–IV	Marital status	Age, race, grade, AJCC stage, Hormone receptor, HER‐2, surgery, chemotherapy, radiotherapy
Luo^75^	2019	USA	Retrospective	19,276	SEER		Women	62.98 (13.75)	Ovarian cancer	I–IV	Marital status	Race, age, histological types, diagnostic year, radiotherapy
Osazuwa‐Peters^29^	2019	USA	Retrospective	460	Hospital Tumor Registry	15	W (26.7) M (73.3)	59.19 (11.33)	Head and neck cancer	Early and late	Marital status	Sex, race, age, alcohol use, insurance status, tobacco use, stage, treatment type, primary site
Qiu^84^	2019	USA	Retrospective	2725	SEER	4	W (43.5) M (56.5)	70.8% ≤50; 29.2 >50	Osteosarcoma	I–IV	Marital status	Age, sex, grade, TNM stage, surgery
Simpson^83^	2019	USA	Retrospective	71,799	SEER	ND	W (23.8) M (76.2)	62.3 (12.1)	Head and neck cancer	I–IV	Marital status	Race, insurance status, stage, site, treatment, age at diagnosis, year of diagnosis, county‐level median income
Yan^80^	2019	USA	Retrospective	1581	SEER	5	W (26.7) M (73.3)	47.7% <60; 52.3% >60	Hepatocellular carcinoma	I–IV	Marital status	Sex, race, age, year of diagnosis, TNM stage, Tumor size, radiotherapy, chemotherapy
Zhai^32^	2019	USA	Retrospective	298,434	SEER	10	W (99.3) M (0.7)	ND	Breast cancer	0‐IV	Marital status	Age, sex, race, stage, grade, surgery, hormone receptor status
Rosiello^45^	2019	USA	Retrospective	11,167	SEER	5	W (31.1) M (68.7)	67.9 (ND)	Non‐metastatic urothelial bladder cancer	0‐IV	Marital status	Age, ethnicity, SES, tumor grade, tumor stage, nodal stage, year of surgery
Zhang^76^	2019	USA	Retrospective	31,895	SEER	5	W (34.9) M (65.1)	55 median (18–64 IQR)	Renal cell carcinoma	I–IV	Marital status	Sex, age, race, tumor size, tumor grade, stage, surgery, insurance status, county level median household income, education, county percentage unemployment
Khan^27^	2019	USA	Retrospective	3579	Institutional cancer registry	10.2	Men	60.4 (7.2)	Prostate cancer	I–II	Marital status	Age, race, comorbidity, log‐transformed PSA, Biopsy Gleason grade
Yang^43^	2019	USA	Retrospective	925	Chi‐Mei medical center Cancer registry	5	W (42.5) M (57.5)	65 (12)	Colon cancer	I–IV	Marital status	Age at diagnosis, lymph node count, stage, grade, perineural invasion, circumferential resection margin, adjuvant treatment
Maas^47^	2020	USA	Retrospective	36,578	Florida Cancer Data System	6–14 years	W (41.3) M (58.7)	62.5 (16.2)	Melanoma	Early and late stage	Marital status	Age at diagnosis, sex, insurance status, race, ethnicity, tobacco use, histology, staging, primary site, geographic are
Rachidi^77^	2020	USA	Retrospective	73,558	SEER	ND	W (45.7) M (54.3)	60.4 (15.8)	Cutaneous melanoma	ND	Marital status	Sex, race, stage, continuous age
Zhou^62^	2020	USA	Retrospective	3947	SEER	ND	W^1^, ^39^ M (60.9)	<older than 50	Gastric neuroendocrine neoplasm	Localized, regional, distant	Marital status	Age at diagnosis, sex, year of diagnosis, ethnicity, grade, tumor stage, size, surgery
Cai^54^	2020	USA	Retrospective	4217	SEER	3.8	W (47.5) M (52.5)	43.0% <60, 57.0% ≥60	Uveal melanoma	I–IV	Marital status	Gender, age, race, diagnosis year, SEER stage, surgery, median household income, registry site
Alyabsi^48^	2021	Saudi Arabia	Retrospective	936	MNG‐HA Cancer registry	5	W (38.3) M (61.7)	46.6% <59, 53.4% >60	Colorectal cancer	Localized, regional, distant metastatic, other	Marital status	Gender, age at diagnosis, stage, pathological grading, tumor site, tumor morphology, chemotherapy status, surgery status, radiotherapy status
Ding^55^	2021	USA	Retrospective	8834	SEER	5	W (18.9) M (81.1)	56.7% ≤65, 43.3 ≥65	Laryngeal cancer	I–IV	Marital status	Sex, age, grade, race, histological type, surgery, AJJC stage, radiotherapy, chemotherapy
Liang^49^	2021	USA	Retrospective	4933	SEER	ND	W (19.6) M (80.4)	38.1% <60, 61.9% ≥60	Liver cancer	I–IV	Marital status	Age, sex, race, grade, AJCC, SEER stage
Xing^50^	2021	USA	Retrospective	3375	SEER	ND	W (41.3) M (58.7)	53.2% ≤60, 46.8 >60	Mycosis fungoides	Localized, regional, distant stage	Marital status	Age, sex, race, T stage
Ai^37^	2021	USA	Retrospective	1344	SEER	ND	W (60.7) M (39.3)	52.9 (15.5)	Medullary thyroid cancer	T1–T4	Marital status	Sex, age, race, tumor stage, nodal stage, metastatic, surgery
Wu^64^	2022	USA	Retrospective	61.928	SEER	5	W (52.4) M (47.6)	40.7% <65, 59.3% ≥65	Lung adenocarcinoma	I–IV	Marital status	Sex, age, race, grade, TNM stage, surgery, radiotherapy, chemotherapy, median household income
Ayaz^25^	2022	USA	Retrospective	1561	SEER	2.6	W (42.0) M (58.0)	55.73 (16.33)	Various	Local, regional, distant stage	Marital status	Age, race, sex, ethnicity, tumor type, primary site, grade, summary stage, number of primary tumors, laterality, use of radiation and use of chemotherapy.

Abbreviations: Cl, confidence interval; HR, hazard ratio; MMR, mortality rate ratio; ND, no data available; NS, non‐significant result; P, prospective l ongitudinal study design; R, retrospective longitudinal study design; RR, relative risk.

Some authors reported only overall survival or cancer specific survival, while some authors reported both. To overcome the issue of unexchangeable results, we performed separate analyses for such reported outcomes. Additionally, the authors of the included studies choose different modes of comparison of marital status. In order to systematically review the published studies, we first needed to categorize the marital status groups. In this review the following marital status groups were therefore described and compared: unmarried versus married, never married versus married, single versus married, divorced/separated versus married, and widowed versus married.

A quality assessment was carried out (performed by KK and JS) following the eight‐item Newcastle‐Ottawa scale for quality assessment of observational studies, which had been adapted for the needs of this review. Two rating categories of the scale, the items “Selection of the non‐exposed cohort” and “Demonstration that outcome of interest was not present at start of study” were not relevant for this review and were therefore excluded. The highest possible score, denoting high study quality, was seven. Studies scoring six and seven were considered of high quality; studies scoring five and four were rated as of moderate quality; and studies scoring lower than four were considered of low quality.

### 2.4 Statistical analyses

Although most of the results are presented descriptively, where possible we used a meta‐analytical approach in order to combine the results of the hazard ratios (HR) in those studies from which this information could be obtained.^14^ The analyses were performed in the software program Review Manager 5.4 (The Nordic Cochrane Centre). We used a random effect model, the DerSimonian and Laird method,^15^ as we expected a certain pattern of variability in the included studies due to different types of cancer, cancer stage, age of the participants, gender, and other factors. In each meta‐analysis, we carried out a heterogeneity analysis.

For interpreting the results of heterogeneity we followed the Cochrane Handbook for Systematic Reviews of Interventions (Version 6.0): (a) 30%–60% may represent moderate heterogeneity; (b) 50%–90%: may represent substantial heterogeneity; (c) 75%–100%: considerable heterogeneity.^16^ In our study, if *I* ^2^ > 75% and *p* < 0.05, and if we had enough studies (*n* ≥ 3), we analyzed the specific subgroup, for example by gender, in order to try to explain the reasons for the heterogeneity.^17^ A *p*‐value of 0.05 or lower was considered statistically significant.

When interpreting clinical relevance, we used the number needed to treat (NNT), which is an indicator of the clinically significant threshold. It applies to the number of patients a clinician would need to treat in order to achieve, on average, one patient with a longer survival.^18^ The NNT was calculated according to the formula: NNT = (1 + HR)/(1−HR). The NNT can be compared with the often‐applied appropriate effect size measure (Cohen's d). Usually single‐digit values for NNT denote a worthwhile difference.^19^ According to Cohen's guidelines,^20^ a NNT of 9 is interpreted as a small effect size, a NNT of 4 as a moderate effect size and a NNT of 3 as a large effect size.

## 3 RESULTS

### 3.1 Study selection

**FIGURE 1:**
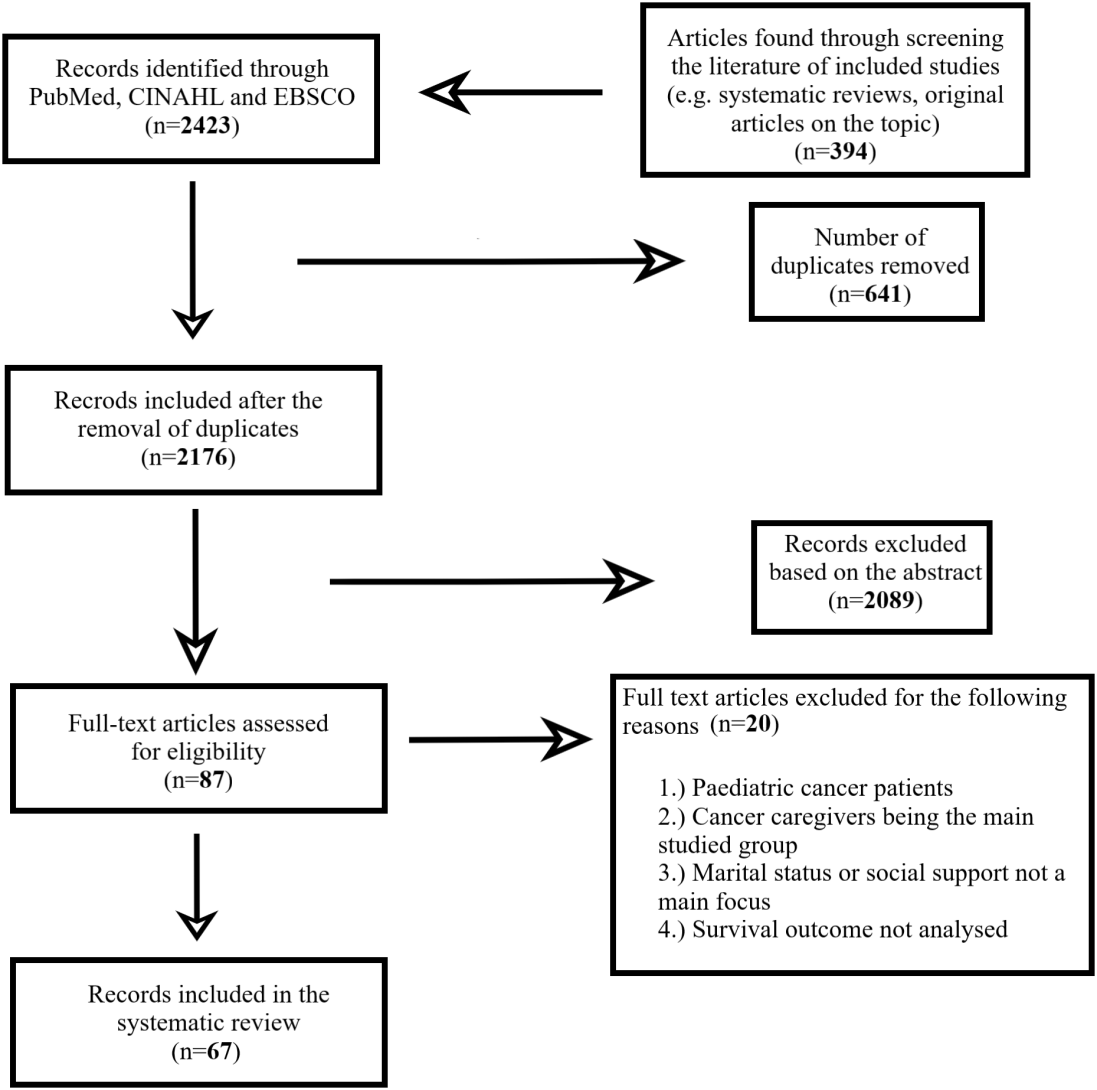
The flowchart of study selection

The search strategy resulted in 2423 articles, plus 394 additional articles included through the screening of the references of the systematic reviews and original articles. After the removal of duplicates and articles that were removed for specific reasons (see Figure 1), 67 articles were left and therefore evaluated in this systematic review.

### 3.2 Characteristics of the included studies (Table 1)

The studies were published between 1987 and 2022. The median follow‐up time ranged from 1 to 19 years. Most of the included studies reported the inclusion of both sexes, but in nine studies there were only female patients and in four studies only male patients. The mean age of the participants in the eligible studies was most often 60 years, although some of the studies reported involving younger cancer patients (*M* < 50 years).

Of the 67 studies, four studies included patients with various cancer sites and did not report results for individual sites; five studies reported results for breast cancer; four studies separately reported results for bladder cancer, gastric cancer, and lung cancer; three studies separately included patients with head and neck cancer and prostate cancer; two separately reported results for cervical cancer, colon cancer, ovarian cancer, pancreatic cancer, gastrointestinal stromal tumor and sarcoma; and 49 studies for other cancer sites (e.g. colorectal cancer, melanoma). Fifty‐five studies included participants with cancer in stages I–IV; five separated cancer patients into “localized, regional and distant stages”; two separated cancer patients into stages 0–IV, into stages I–II and into “early and late” stages. One study separately included participants with cancer into stages II–IV, into stages “T4, N1 or M1”, into stages IB2‐IVA and into stages T1–T4. In the four remaining studies, no data on the stage of cancer were provided (see Table 1).

On assessing study quality using the Newcastle‐Ottawa scale, we found that 56 studies were deemed to be of high quality and 11 were deemed to be of moderate quality.

### 3.3 Adjusted analysis

All the articles except one^21^ reported an adjusted analysis as their main outcome. Most commonly they adjusted the analysis for demographic characteristics such as age, gender, and race. Most articles also adjusted the analysis for tumor stage, type of therapy, tumor grade, type of surgery and other less common variables (e.g. tobacco use, household income, geographic area; see Table 1).

### 3.4 Analysis of the comparison between unmarried and married patients

#### 3.4.1 Overall survival

Sixteen articles reported a sub‐category which compared the overall survival of unmarried patients to married ones, of which all but one^22^ reported significant difference between them. The association was found in three studies on patients with mixed types of cancer,^23^, ^24^, ^25^ two studies on patients with prostate cancer^26^, ^27^; and one study each in patients with bladder,^28^ head and neck,^29^ testicular,^30^ pancreatic,^31^ breast,^32^, ^33^ kidney,^34^ esophageal,^35^ gastric^36^ and medullary thyroid cancer.^37^

**FIGURE 2:**
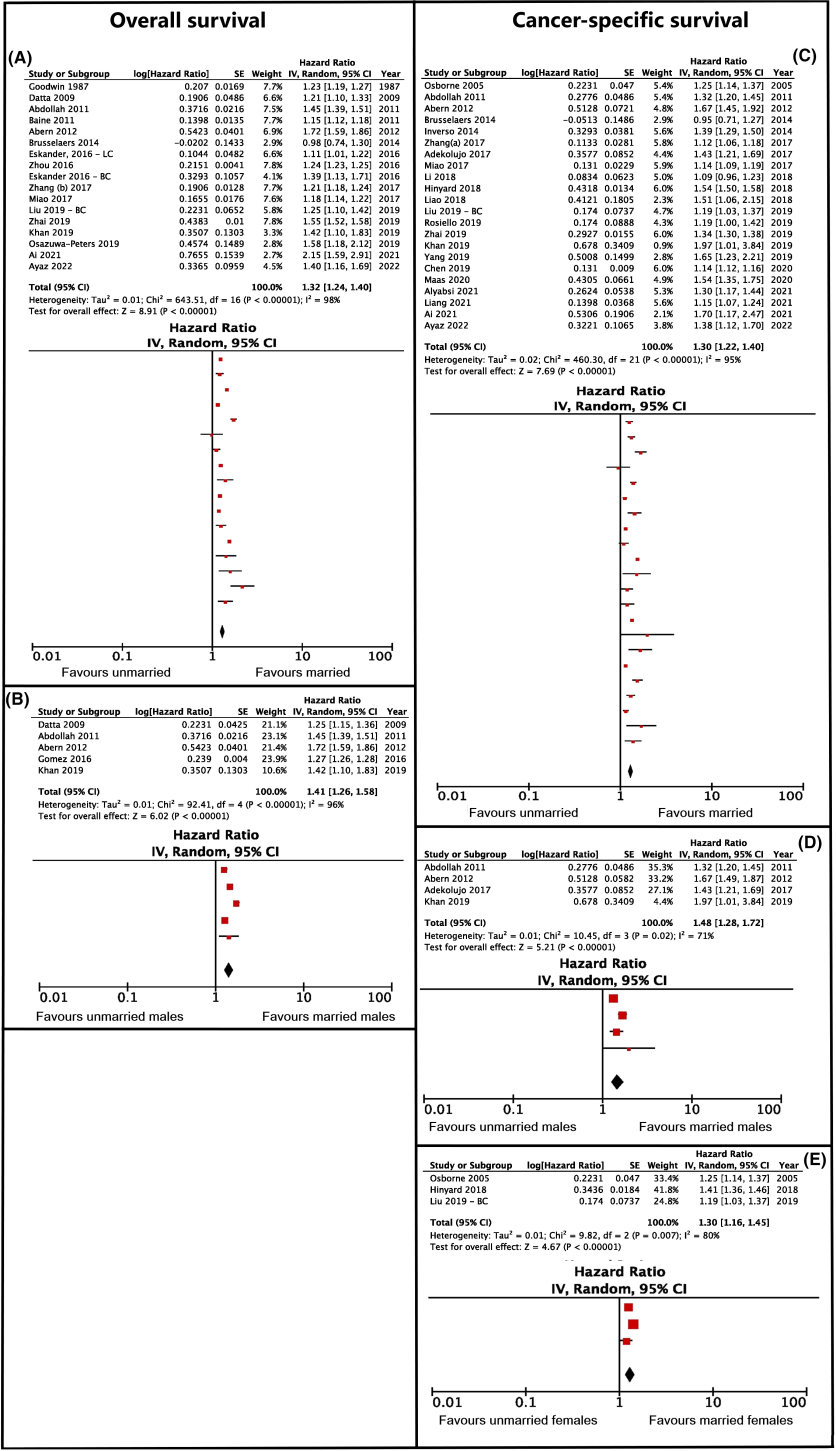
Overall and cancer‐specific survival hazard ratios comparing unmarried and married cancer patients. (A, C) Pertain to overall survival, (B, D, E) pertain to cancer‐specific survival. (A) Depicts the analysis without subanalyses by gender, (B) depicts the results of the subanalysis of overall survival for males only. (C) Shows the results of the main analysis, while (D, E) show the results of subanalyses for males and females, respectively.

The meta‐analysis showed that the total hazard ratio for unmarried versus married patients was 1.32 with a confidence interval of 1.24–1.40 (*p* < 0.001) and an NNT value of 7 (see Figure 2A). Due to the high heterogeneity in the results (*I* ^2^ = 98%), the studies were categorized according to gender (see Figure 2B). Following this, the total hazard ratio of unmarried versus married men amounted to 1.41 with a confidence interval of 1.26–1.58 (*p* < 0.001) and an NNT value of 6. However, the heterogeneity was still not decreased, meaning that there are other variables contributing to this result (*I* ^2^ = 96%). It was not possible to calculate a total hazard ratio of unmarried versus married women, due to the small number of studies that performed gender analysis (*n* = 2).

#### 3.4.2 Cancer‐specific survival

Twenty‐two studies compared cancer specific survival in unmarried and married cancer patients, of which all but two^22^, ^38^ reported that married patients had a higher cancer‐specific survival rate than unmarried patients. The association was found in five studies on patients with breast cancer,^21^, ^32^, ^33^, ^39^, ^40^ two studies on patients with prostate cancer^26^, ^27^; and one study each in patients with testicular,^30^ head and neck,^41^ gastric,^42^ kidney,^34^ colon,^43^ oral cavity,^44^ non‐metastatic urothelial bladder cancer,^45^ non‐small cell lung cancer,^46^ melanoma,^47^ medullary thyroid,^37^ colorectal^48^ and liver cancer,^49^ and in one study with various cancer types.^25^

The meta‐analysis showed that the total hazard ratio for unmarried versus married patients was 1.30, with a confidence interval of 1.22–1.40 (*p* < 0.001) and an NNT value of 8 (see Figure 2C). Due to the high heterogeneity in the results (*I* ^2^ = 95%), the studies were categorized according to gender (see Figure 2D,E). Following this, the total hazard ratio of unmarried versus married women rose to 1.30 with a confidence interval of 1.16–1.45 (*p* < 0.0001) and an NNT value of 8. The total hazard ratio of unmarried versus married men amounted to 1.48 with a confidence interval of 1.28–1.72 (*p* < 0.001) and an NNT value of 5. A statistical comparison between women and men showed no significant difference between the groups on cancer survival (*z* = 1.48; *p* = 0.20). The heterogeneity in both sub‐group analyses was still high (*I* ^2^ = 93%, *I* ^2^ = 71%); however, due to an insufficient number of studies (*n* = 3–4 in each group), heterogeneity could not be further analyzed.

### 3.5 Analysis of the comparison between single and married cancer patients

#### 3.5.1 Overall survival

**FIGURE 3:**
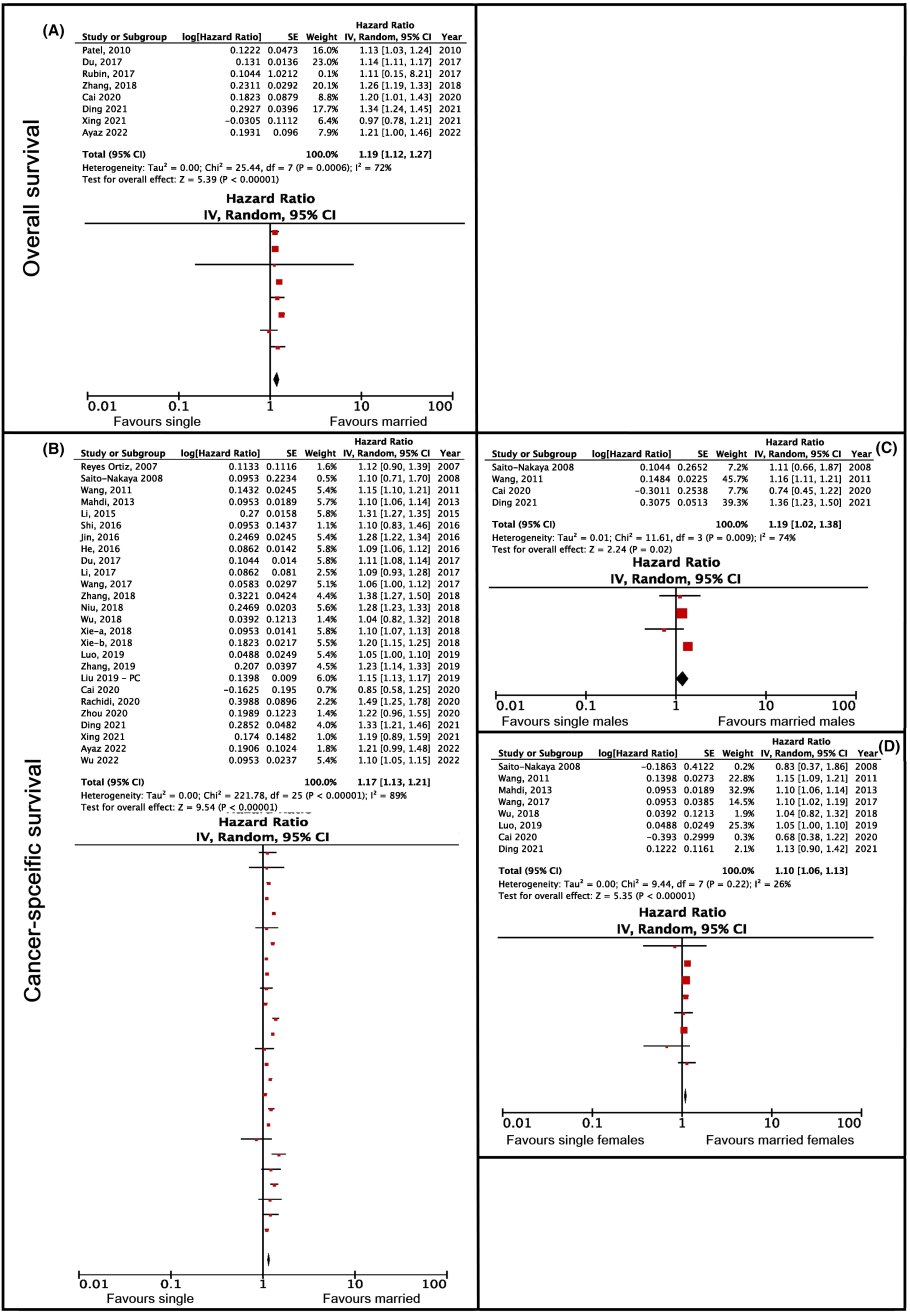
Overall and cancer‐specific survival hazard ratios comparing single and married cancer patients. (A) Pertains to overall survival, (B–D) pertain to cancer‐specific survival. (A) Shows the main analysis, without further subgroup analyses. (B) Shows the main analysis, while (C, D) represent the results of subanalyses by gender, for males and females, respectively.

The sub‐category single versus married patients was compared in eight studies. All but two^50^, ^51^ reported that married patients had higher overall survival rates than single patients. The association was found in studies on patients with cervical,^52^ esophageal,^53^ uveal melanoma,^54^ laryngeal cancer,^55^ soft tissue sarcoma^56^ and in one study with various cancer types.^25^ The meta‐analysis showed that the hazard ratio was 1.19 with a confidence interval of 1.12–1.27 (*p* < 0.005) and an NNT value of 14 (Figure 3A). The heterogeneity was found to be substantial (*I* ^2^ = 67%); however, it was not possible to carry out further analysis.

#### 3.5.2 Cancer‐specific survival

The sub‐category single versus married patients was compared in twenty‐six studies. Nine studies^25^, ^50^, ^54^, ^57^, ^58^, ^59^, ^60^, ^61^, ^62^ did not find the association between the groups, while the rest reported that married patients had higher cancer‐specific survival rates than single patients. The association was found in studies on patients with non‐small cell lung cancer,^63^ lung adenocarcinoma,^64^ pancreatic cancer,^65^ epithelial ovarian cancer,^66^ colorectal cancer,^67^ gastric cancer,^68^ liver cancer,^69^ esophageal cancer,^53^ prostate cancer,^70^ renal cancer,^71^ bladder urothelial cancer,^72^ astrocytoma,^73^ ovarian cancer,^75^ renal cell carcinoma,^76^ cutaneous melanoma,^77^ laryngeal cancer,^55^ and soft tissue sarcoma.^56^

The meta‐analysis showed that the hazard ratio was 1.17 with a confidence interval of 1.13–1.21 (*p* < 0.001) and an NNT value of 13 (Figure 3B). Due to the high heterogeneity in the results (*I* ^2^ = 89%), the studies were categorized according to gender (see Figure 3C,D). The total hazard ratio of single versus married women was 1.10, with a confidence interval of 1.06–1.13 (*p* < 0.001) and an NNT value of 21; the total risk of single versus married men amounted to 1.19, with a confidence interval of 1.02–1.38 (*p* < 0.005) and a NNT value of 12. Sex differences between single versus unmarried patients did not show any statistically significant differences between women and men (z = 0.97; *p* > 0.05). The heterogeneity analysis was substantial in men (*I* ^2^ > 74%), but not in women (I^2^ > 26%); however, due to the small number of studies (*n* = 3) and the lack of variables, it was not possible to carry out further sub‐analysis.

### 3.6 Analysis of the comparison between never‐married and married patients

#### 3.6.1 Overall survival

**FIGURE 4:**
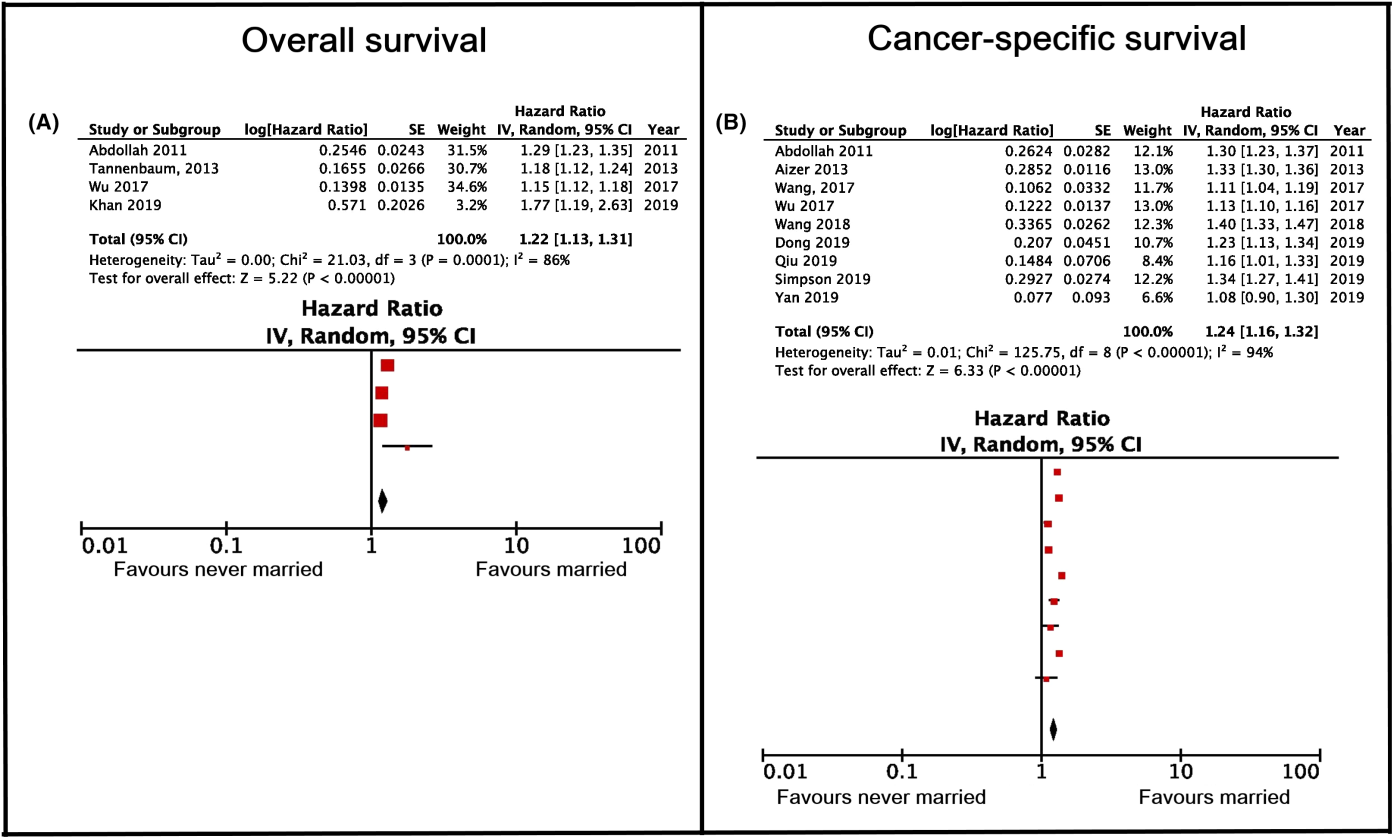
Overall and cancer‐specific survival hazard ratios comparing never married and married cancer patients. (A) Pertains to overall survival and (B) pertains to cancer‐specific survival.

The sub‐category never‐married versus married patients was compared in four studies. All the studies reported that married patients had higher overall survival rates than never‐married patients. The association was found in two studies of patients with prostate cancer,^26^, ^27^ and in studies with lung^78^ and non‐small cell lung cancer.^79^ The meta‐analysis showed that the hazard ratio was 1.22 with a confidence interval of 1.13–1.31 (*p* < 0.001) and an NNT value of 10 (Figure 4A). The heterogeneity was substantial (*I* ^2^ = 86%); however, due to the small number of studies, it was not possible to perform additional analyses.

#### 3.6.2 Cancer‐specific survival

The sub‐category never‐married versus married patients was compared in nine studies. All the studies except one,^80^ reported that married patients had higher cancer‐specific survival rates than never‐married patients. The association was found in studies on patients with prostate,^26^ renal,^71^ rectal,^81^ non‐small cell lung,^79^ endometrial^82^ and head and neck cancer,^83^ osteosarcoma^84^ and in one study with patients with various cancer types.^85^ The meta‐analysis showed that the hazard ratio was 1.24 with a confidence interval of 1.16–1.32 (*p* < 0.001) and an NNT value of 9 (Figure 4B). The heterogeneity was substantial (*I* ^2^ = 91%), but analysis with further subgroup division was not possible due to the small number of studies.

### 3.7 Analysis of the comparison between divorced/separated and married patients

#### 3.7.1 Overall survival

**FIGURE 5:**
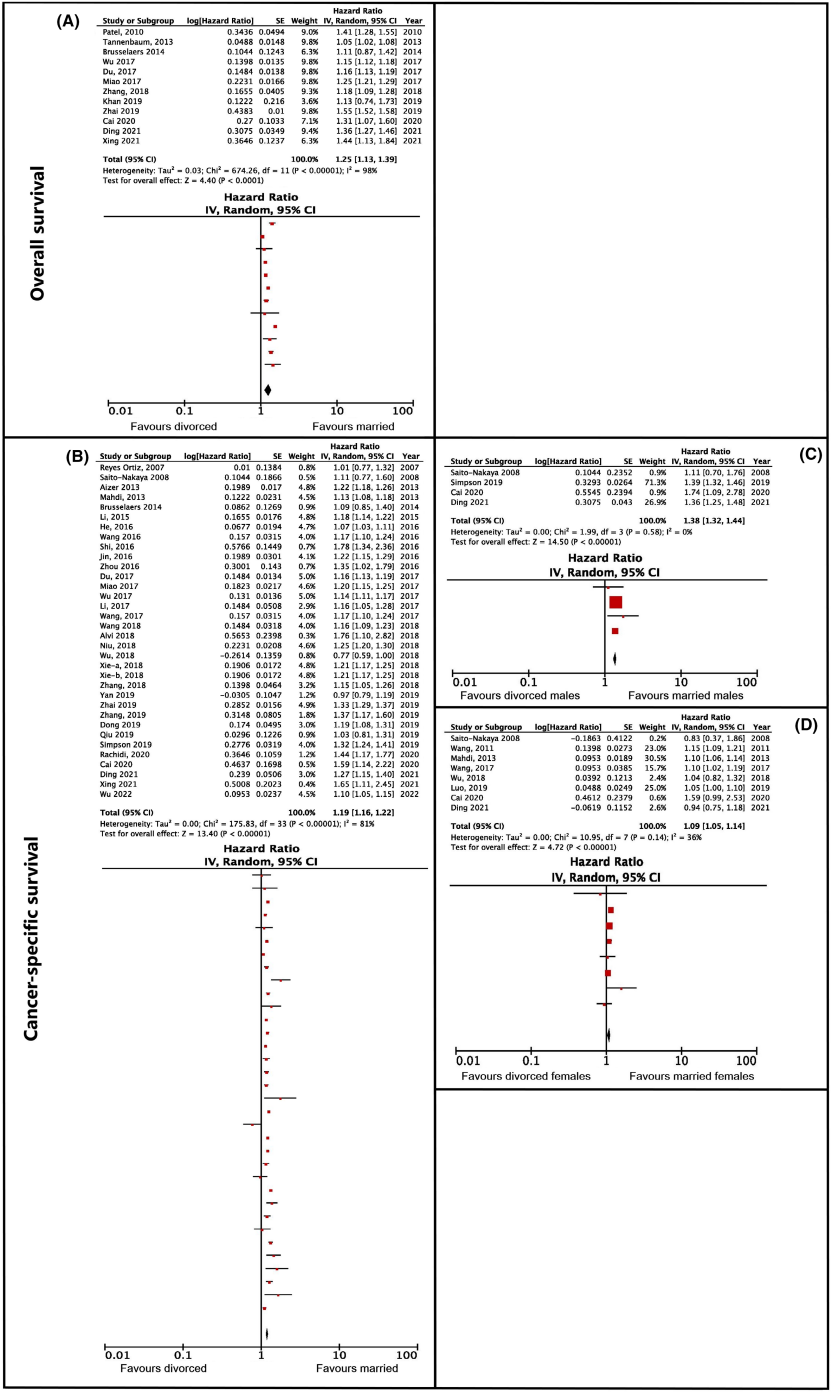
Overall and cancer‐specific survival hazard ratios comparing divorced and married cancer patients. (A) Pertains to overall survival, (B–D) pertain to cancer‐specific survival. (A) Shows the main analysis, without further subgroup analyses. (B) Shows the main analysis, while (C, D) represent the results of subanalyses by gender, for males and females, respectively.

The sub‐category of divorced/separated versus married people was compared in 12 studies. All but two^22^, ^27^ reported that married patients had a higher overall survival rate than divorced/separated ones. The association between marital status and better survival was found in studies on patients with cervical,^52^ lung,^78^ esophageal,^53^ kidney,^34^ vulvar,^61^ breast^32^ and laryngeal cancer.^55^ The association was also found in studies of patients with uveal melanoma,^54^ soft tissue sarcoma^56^ and mycosis fungoides.^50^ The meta‐analysis showed that the total hazard ratio was 1.25, with a confidence interval of 1.13–1.39 (*p* < 0.001) and an NNT value of 9 (Figure 5A). The heterogeneity was substantial (*I* ^2^ = 98%); however, it was not possible to perform additional analyses.

#### 3.7.2 Cancer‐specific survival

The sub‐category of divorced/separated versus married people was compared in 34 studies, of which six studies^22^, ^57^, ^61^, ^63^, ^80^, ^84^ did not find an association between the groups. Twenty‐eight reported that married patients had a higher cancer‐specific survival rate than divorced or separated patients. The association between marital status and better survival was found in two studies with patients with gastric cancer,^36^, ^68^ and in one study each on patients with epithelial ovarian,^66^ esophageal,^53^ colorectal,^67^ liver,^69^ renal,^71^ thyroid,^58^ kidney,^34^ gallbladder,^60^ endometrial,^82^ head and neck,^83^ breast,^32^ rectal,^81^ lung,^79^ pancreatic^86^ and laryngeal cancer.^55^ The association was also found in one study each on patients with bladder urothelial carcinoma,^72^ astrocytoma,^73^ spinal cord tumours,^87^ glioblastoma multiforme,^74^ renal cell carcinoma,^76^ cutaneous melanoma,^77^ uveal melanoma,^54^ laryngeal cancer,^55^ lung adenocarcinoma^64^ and mycosis fungoides,^50^ and in one study on patients with various cancer types.^85^

The meta‐analysis showed that the total hazard ratio was 1.19, with a confidence interval of 1.16–1.23 (*p* < 0.001) and an NNT of 12 (Figure 5B). Due to the high heterogeneity in the results (*I* ^2^ = 82%), the studies were sub‐analyzed according to gender (see Figures 3D and 5C). Following this, the total hazard ratio of divorced/separated versus married women was 1.09, with a confidence interval of 1.05–1.14 (*p* > 0.001) and an NNT value of 23. The total hazard ratio of men amounted to 1.38, with a confidence interval of 1.32–1.44 (*p* > 0.001) and an NNT value of 6. The analysis of sex differences between married versus divorced/separated found statistically significant differences between the two groups (*z* = 7.91; *p* < 0.001). The heterogeneity could be explained by gender in the sub‐group of divorced/separated versus married men (*I* ^2^ = 0%); however, in the sub‐analysis of female patients it was found to be small‐moderate (*I* ^2^ = 36%).

### 3.8 Analysis of the comparison between widowed and married patients

#### 3.8.1 Overall survival

**FIGURE 6:**
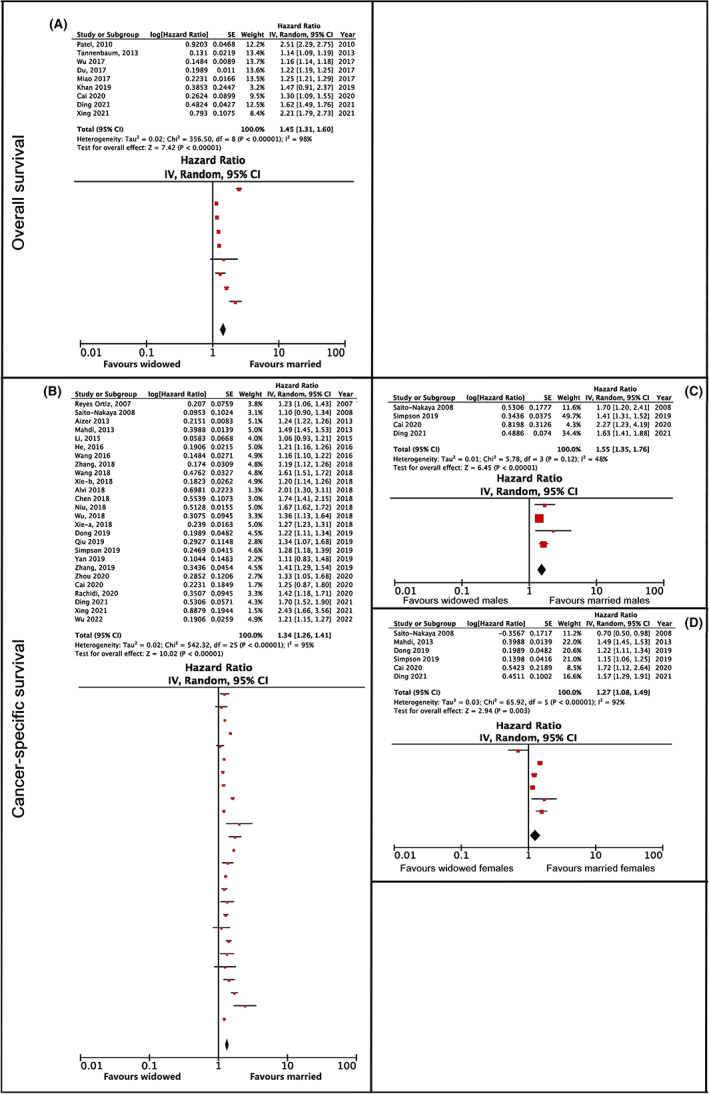
Overall and cancer‐specific survival hazard ratios comparing widowed and married cancer patients. (A) Pertains to overall survival, (B–D) pertain to cancer‐specific survival. (A) Shows the main analysis, without further subgroup analyses. (B) Shows the main analysis, while panels C and D represent the results of subanalyses by gender, for males and females, respectively.

The sub‐category of widowed versus married patients appeared in 11 studies. All the studies except one^27^ reported that married patients had a better overall survival than widowed patients. The association was found in studies involving patients with cervical,^52^ lung,^78^ kidney,^34^ esophageal,^53^ laryngeal,^55^ and non‐small cell lung cancer.^79^ The association was also found in studies of patients with uveal melanoma,^54^ soft tissue sarcoma,^56^ gastrointestinal stromal tumor^88^ and mycosis fungoides.^50^ The meta‐analysis showed that the total hazard ratio was 1.45, with a confidence interval of 1.31–1.60 (*p* < 0.001) and an NNT value of 5 (see Figure 6A). The heterogeneity was found to be considerable (*I* ^2^ = 98%); however, no further analysis was possible.

#### 3.8.2 Cancer‐specific survival

The category widowed versus married patients appeared in 26 studies. All the studies except two^63^, ^67^ reported that married patients had a better cancer‐specific survival than widowed patients. The association was found in studies involving patients with melanoma,^57^ epithelial ovarian cancer,^66^ liver cancer,^69^ renal cancer,^86^ glioblastoma multiforme,^74^ spinal cord tumours,^87^ gastrointestinal stromal tumor,^88^ bladder urothelial carcinoma,^72^ vulvar cancer,^61^ astrocytoma,^73^ endometrial cancer,^82^ osteosarcoma,^84^ head and neck cancer,^83^ hepatocellular carcinoma,^80^ renal cell carcinoma,^76^ cutaneous melanoma,^77^ gastric neuroendocrine neoplasm,^62^ uveal melanoma,^54^ laryngeal cancer,^55^ lung adenocarcinoma,^64^ mycosis fungoides^50^ and rectal cancer,^81^ soft tissue sarcoma^56^ and in one study of patients with various types of cancer.^85^

The meta‐analysis showed that the total hazard ratio was 1.34, with a confidence interval of 1.26–1.41 (*p* < 0.001) and an NNT value of 7 (see Figure 6B). Due to the high heterogeneity in the results (*I* ^2^ = 95%), the studies were categorized according to gender (Figure 6C,D). Following this, the total hazard ratio of widowed versus married women amounted to 1.27, with a confidence interval of 1.08–1.49 (*p* < 0.001) and an NNT value of 8. The total hazard ratio of widowed versus married men was 1.55, with a confidence interval of 1.35–1.76 (*p* < 0.001) and an NNT value of 5. Statistical comparisons between genders showed no observed difference in cancer mortality between men and women (*z* = 1.82; *p* > 0.05). The heterogeneity was still found to be considerable in the sub‐analysis of female patients (*I* ^2^ = 92%), and moderate in male patients (*I* ^2^ = 48%).

## 4 DISCUSSION

We systematically reviewed and performed a meta‐analysis of non‐interventional studies that explored the association between marital status and cancer‐specific and overall survival. We did not limit ourselves to the type or the stage of cancer in selecting our range of studies, so we obtained a comprehensive overview of the presented topic. Here we present data from 70 articles that reported on the association between marital status and survival.

Our meta‐analysis showed that, compared to unmarried patients, being married was significantly associated with better overall survival (NNT of 7 with a small to moderate effect, *p* < 0.001) and cancer‐specific survival (NNT of 8 with a small to moderate effect, *p* < 0.001). Additionally, we found that married patients had better overall and cancer‐specific survival when compared to single patients (an NNT of 14 with a small effect, *p* < 0.005, and an NNT of 13 with a small effect, *p* < 0.001, respectively); never‐married patients (an NNT of 10 with a small effect, *p* < 0.001, and an NNT of 9 with a small to moderate effect, *p* < 0.001, respectively); divorced/separated patients (an NNT of 9 with a small effect, *p* < 0.001, and an NNT of 12 with a small effect, *p* < 0.001, respectively); and widowed patients (an NNT of 5 with a small to moderate effect, *p* < 0.001 in overall and an NNT of 7 with a small to moderate effect, *p* < 0.001, in cancer‐specific survival). The statistics on gender group differences showed that the difference between the genders was statistically significant only between divorced/separated versus married men and women (*p* < 0.0001). Although the hazard ratio was higher in all the sub‐analyses for men compared to women, a statistically significant gender difference was not found in any of the other sub‐analyses.

The highest clinical significance was found when comparing married and widowed cancer patients' overall (HR 1.45, 95% Cl 1.31–1.60, *p* < 0.001, NNT of 5) and cancer‐specific survival (HR 1.34, 95% Cl 1.26–1.41, *p* < 0.001, NNT of 7) (Figure 6A,B). Besides the problems, threats and burdens associated with the illness, these patients must also face the loss of an important person, which may have a negative impact on their health.^89^ Being a widow or widower is usually accompanied by high emotional stress or grief, a reduction in their social network, and at the same time the loss of the material support provided by a spouse. Widows and widowers accept chemotherapy less frequently than those who are married, and are more likely to find treatment in healthcare facilities of lower quality.^90^ Studies have shown that widowed individuals have a poorer immune response: a poorer lymphocyte response in the peripheral blood regions^91^, ^92^ and reduced activity of natural killer cells^93^, ^94^ which play a key role in the identification and removal of cancer cells.^11^

The second highest clinical significance was when comparing married versus unmarried patients' overall (HR 1.32, 95% CI 1.24–1.40; *p* > 0.001, NNT of 7) and cancer‐specific survival (HR 1.30, 95% CI 1.22–1.40; *p* > 0.001, NNT of 8) (Figure 2A,C). This result can be interpreted through various mechanisms which, in addition to psychosocial factors, also include economic and environmental factors.^12^ Having a partner or spouse is associated with a healthier lifestyle,^95^ a greater chance of discovering the disease at an earlier stage and deciding on active treatment,^96^ higher financial income,^97^ and better mental health.^98^ Cancer is a great stressor for the person affected, so emotional support, which the spouse can offer in a specific way, can help to reduce the negative effects of stress, which can lead to better outcomes of the treatment itself.^8^ The presence of a loving and caring partner is also associated with an increased release of the hormone oxytocin, which can inhibit the growth of cancer cells through indirect and direct mechanisms.^11^

A smaller clinical significance (NNT >8) was found in divorced/separated patients (overall: HR 1.25, 95% CI 1.13–1.39, *p* < 0.001; and cancer‐specific survival: HR 1.19, 95% CI 1.16–1.23, *p* < 0.001; see Figure 5A,B); single patients (overall: HR 1.19, 95% CI 1.12–1.27, *p* < 0.005; and cancer‐specific survival: HR 1.17, 95% CI 1.13–1.21, *p* < 0.001; see Figure 3A,B); and never‐married patients (overall: HR 1.22, 95% CI 1.13–1.31, *p* < 0.001; and cancer‐specific survival: HR 1.24, 95% CI 1.16–1.32, *p* < 0.001; see Figure 4A,B) compared to married cancer patients. Although divorce from a spouse can be a stressful event, it is possible that patients were able to build their own social network, thus replacing the support that a partner or a spouse could have offered them. Single and never‐married patients have not been exposed to a stressful event such as separation or the death of their spouse, as experienced by the widowed patients, which could explain the lower clinical significance found in this group.

While being unmarried, single, divorced/separated, or widowed conferred a higher risk of cancer‐specific survival for men than for women, the difference between the genders was statistically significant only for the sub‐analysis of divorced/separated versus married group. This could be explained by the fact that women are more likely to encourage their spouse to have a health‐beneficial lifestyle than vice versa.^8^ Married male cancer patients have reported significantly lower levels of psychological distress and higher psychological support from their spouse, which may have resulted in a better cancer prognosis.^9^ It has also been suggested that men gain more social benefits from marriage, while women gain more financial benefits.^99^ Women are expected to benefit more from a large social network than from marriage alone, at least according to the results of the comparison of large versus small social networks found in two studies,^100^, ^101^ and the finding that unmarried women have longer survival rates than unmarried men, as found in this study.

Our conclusions are somewhat different when comparing our study to the earlier two previous meta‐analyses conducted on social support and cancer survival. The first meta‐analysis published in 2010,^12^ including 87 studies, reported that the worst survival rate was observed in never‐married patients compared to the married group. While our study found significant differences when comparing the never‐married versus married group, the greatest differences were found in the general unmarried group and the widowed group (as compared to the married group). The differences in the findings could be attributed to the fact that our study was conducted 12 years later when couples are less likely to decide to get married than they used to be. Being unmarried does not necessarily mean that they do not have a partner. Compared to the newer study,^13^ carried out in 2018 and examining the results of 21 studies, our study confirms that divorced/separated men had a higher risk of cancer mortality compared to the mortality rate found in women. Future prospective studies should consider these findings when planning appropriate sample size, in order to be able to perform additional gender‐adjusted analyses.

Although much care was taken to limit the confounders of our analyses and literature review, some limitations are nonetheless present in this study. The first is that some of the included studies did not provide data on the age, gender, and stage of cancer of the patients, making it difficult to determine whether the observed associations were moderated by the demographic and clinical characteristics of cancer patients. Additionally, the category of unmarried patients was quite heterogeneous and, when referred to in studies without additional explanation, can be hard to interpret, as it could mean the patients were divorced, separated, never‐married, single, widowed or any combination thereof.

Despite these limitations, 63 articles represent a large portion of original research on the association between marital status and the survival of cancer patients, spanning more than three decades. Further strengths of the present systematic review are the categorization of the various subgroups of unmarried patients and the categorization of overall and cancer‐specific survival, increasing the accuracy of the conclusions made. Furthermore, where available, a separate analysis for men and women was carried out to test for possible moderator variables.

## 5 CONCLUSION

Our systematic review of the literature showed that being married is associated with improved overall and cancer‐specific survival. The main conclusion is that of the different subgroups of unmarried patients, the widowed are the group with the shortest survival rate, possibly reflecting diminished social contact and the effects of stress and loss on the health of patients. To elucidate the details of this association and determine the contributing factors that moderate the link between marital status and cancer survival, further research into the bodily and psychological processes following the loss of a spouse should be carried out. Additionally, the subgroup analysis by gender showed that divorced/separated men have the worst survival rate when compared to the female cancer patients. This review carries important clinical and research implications, where clinicians can benefit by being aware of the effects of marital status on cancer treatment, enabling them to more easily identify patients in need of comprehensive intervention. The research community can benefit from the findings of this review and meta‐analysis by taking into account the differences in the subgroups of unmarried patients when designing further studies, as well as the differences in the effects of marital status on men and women.

## AUTHOR CONTRIBUTIONS

The contributions of the authors are as follows (as per the CRediT taxonomy); K.K.: Conceptualization, data curation, investigation, writing/original draft preparation, and writing/review and editing. S.M.: Conceptualization, data curation, formal analysis, funding acquisition, methodology, visualization, writing/original draft preparation, and writing/review and editing. J.S.: Data curation, investigation, visualization, writing/review and editing. Z.K.K.: Supervision, project administration, funding acquisition. M.D.: Funding acquisition, supervision, project administration. D.S.: Project administration, supervision, writing/review and editing. G.D. Writing/review and editing, project administration, supervision.

## FUNDING INFORMATION

Spela Mirosevic and Jakob Sajovic acknowledge funding by the Slovenian Research Agency via Programs MR‐39262 and P3‐0293(B), respectively.

## CONFLICT OF INTEREST

The authors declare that the research was conducted in the absence of any commercial or financial relationships that could be construed as a potential conflict of interest.

## ETHICAL STATEMENT

Ethics approval was not required for this study.

## Supporting information


Table S1

Table S2

## Data Availability

The data that support the findings of this study are available from the corresponding author upon reasonable request.
