# Supplementary material for: Marital status and survival in cancer patients: A systematic review and meta‐analysis
Source: Cancer Med. 2022 Jul 4;12(2):1685–708. doi: 10.1002/cam4.5003 (PMC9883406; doi:10.1002/cam4.5003)
Supplement: Supplementary file 1 — Table S1 [file CAM4-12-1685-s001.docx]

**Table S1.** Example of search strategy presented for EBSCO and PubMed.

| **EBSCO** | **PubMED** |
| --- | --- |
| (((MH "Neoplasms") OR ((MH "Cancer Survivors") OR (Cancer survival) OR (CSS))) AND ((MH "Survival") OR (MH "Survival Analysis") OR (MH "Kaplan-Meier Estimate") OR (MH "Mortality") OR (Survival)) AND ((MH "Marital Status") OR (MH "Social Support") OR (Marital status))) NOT ( meta-analysis or systematic review ) NOT tissue | (("cancer s"[All Fields] OR "cancerated"[All Fields] OR "canceration"[All Fields] OR "cancerization"[All Fields] OR "cancerized"[All Fields] OR "cancerous"[All Fields] OR "neoplasms"[MeSH Terms] OR "neoplasms"[All Fields] OR "cancer"[All Fields] OR "cancers"[All Fields]) AND ("mortality"[MeSH Subheading] OR "mortality"[All Fields] OR "survival"[All Fields] OR "survival"[MeSH Terms] OR "survivability"[All Fields] OR "survivable"[All Fields] OR "survivals"[All Fields] OR "survive"[All Fields] OR "survived"[All Fields] OR "survives"[All Fields] OR "surviving"[All Fields]) AND (("marital status"[MeSH Terms] OR ("marital"[All Fields] AND "status"[All Fields]) OR "marital status"[All Fields]) AND ("social support"[MeSH Terms] OR ("social"[All Fields] AND "support"[All Fields]) OR "social support"[All Fields]))) AND ((fft[Filter])) |
