# Supplementary material for: Marital status and survival in cancer patients: A systematic review and meta‐analysis
Source: Cancer Med. 2022 Jul 4;12(2):1685–708. doi: 10.1002/cam4.5003 (PMC9883406; doi:10.1002/cam4.5003)
Supplement: Supplementary file 2 — Table S2 [file CAM4-12-1685-s002.docx]

Table S2. Quality of studies assessed by The Newcastle Ottawa scale for quality assessment of observational studies

| Authors | Selection | | | | Comparability | Outcome | | | Score |
| --- | --- | --- | --- | --- | --- | --- | --- | --- | --- |
|  | Representativeness of studies | Selection of the nonexposed cohort | Ascertainment of exposure | Outcome of interested not present at start | Study controls for other variables | F/U long enough for outcome to occur | Complete F/U of all subjects accounted for | Subjects lost to F/U unlikely to introduce bias |  |
| Alvi et al. 2018 | / | N/A | * | N/A | ** | * | * | * | 6/7 |
| Chen et al. 2018 | / | N/A | * | N/A | ** | * | * | * | 6/7 |
| Chen et al. 2019 | * | N/A | * | N/A | ** | * | * | * | 7/7 |
| Dong et al. 2019 | / | N/A | * | N/A | ** | * | * | * | 6/7 |
| Liao et al. 2018 | / | N/A | * | N/A | ** | * | * | * | 6/7 |
| Liu et al. 2019 – BC | / | N/A | * | N/A | ** | * | / | / | 4/7 |
| Liu et al. 2019 – PC | / | N/A | * | N/A | ** | * | * | * | 6/7 |
| Luo et al. 2019 | * | N/A | * | N/A | ** | * | * | * | 7/7 |
| Maas et al. 2020 | * | N/A | * | N/A | ** | * | / | / | 5/7 |
| Niu et al. 2018 | * | N/A | * | N/A | ** | * | * | * | 7/7 |
| Osazuwa-Peters et al. 2019 | * | N/A | * | N/A | ** | * | / | / | 5/7 |
| Qiu et al. 2019 | / | N/A | * | N/A | ** | * | * | * | 6/7 |
| Rachidi et al. 2020 | * | N/A | * | N/A | ** | * | / | / | 5/7 |
| Rosiello et al. 2019 | / | N/A | * | N/A | ** | * | * | * | 6/7 |
| Simpson et al. 2019 | * | N/A | * | N/A | ** | * | * | * | 7/7 |
| Wu et al. 2018 | / | N/A | * | N/A | ** | * | * | * | 6/7 |
| Xie et al. 2018 – a | / | N/A | * | N/A | ** | * | * | * | 6/7 |
| Xie et al. 2018 – b | / | N/A | * | N/A | ** | * | * | * | 6/7 |
| Yan et al. 2019 | / | N/A | * | N/A | ** | * | * | * | 6/7 |
| Yang et al. 2020 | * | N/A | * | N/A | ** | * | * | * | 7/7 |
| Zhai et al. 2019 | / | N/A | * | N/A | ** | * | * | * | 6/7 |
| Zhang et al. 2018 | / | N/A | * | N/A | ** | * | * | * | 6/7 |
| Zhang et al. 2019 | * | N/A | * | N/A | ** | * | * | * | 7/7 |
| Zhou et al. 2020 | / | N/A | * | N/A | ** | * | * | * | 6/7 |
| Li et al. 2018 | / | N/A | * | N/A | ** | * | * | * | 6/7 |
| Goodwin et al. 1987 | / | N/A | * | N/A | * | * | * | * | 5/7 |
| Osborne et al. 2005 | / | N/A | * | N/A | ** | * | * | * | 6/7 |
| Reyes Ortiz et al. 2007 | * | N/A | * | N/A | ** | * | * | * | 7/7 |
| Saito-Nakaya et al. 2008 | / | N/A | * | N/A | ** | * | * | * | 6/7 |
| Datta et al. 2009 | / | N/A | * | N/A | ** | * | * | * | 6/7 |
| Patel et al. 2010 | / | N/A | * | N/A | ** | * | * | * | 6/7 |
| Abdollah et al. 2011 | * | N/A | * | N/A | ** | * | * | * | 7/7 |
| Baine et al. 2011 | * | N/A | * | N/A | ** | * | * | * | 7/7 |
| Wang et al. 2011 | / | N/A | * | N/A | ** | * | * | * | 6/7 |
| Abern et al. 2012 | * | N/A | * | N/A | ** | * | * | * | 7/7 |
| Tannenbaum et al. 2013 | * | N/A | * | N/A | ** | * | * | * | 7/7 |
| Aizer et al 2013 | * | N/A | * | N/A | ** | * | * | * | 7/7 |
| Mahdi et al. 2013 | / | N/A | * | N/A | ** | * | * | * | 6/7 |
| Brusselaers et al. 2014 | * | N/A | * | N/A | ** | * | * | * | 7/7 |
| Inverso et al. 2014 | * | N/A | * | N/A | ** | * | * | * | 7/7 |
| Li et al.  2015 | / | N/A | * | N/A | ** | * | * | * | 6/7 |
| Wang et al. 2016 | * | N/A | * | N/A | ** | * | * | * | 7/7 |
| Zhou et al. 2016 | / | N/A | * | N/A | ** | * | / | / | 4/7 |
| Eskander et al. 2016 | / | N/A | * | N/A | ** | * | / | / | 4/7 |
| Shi et al. 2016 | * | N/A | * | N/A | ** | * | * | * | 7/7 |
| Jin et al.  2016 | / | N/A | * | N/A | ** | * | * | * | 6/7 |
| He et al.  2017 | * | N/A | * | N/A | ** | * | * | * | 7/7 |
| Adekolujo et al. 2017 | * | N/A | * | N/A | ** | * | * | * | 7/7 |
| Du et al.  2017 | * | N/A | * | N/A | ** | * | * | * | 7/7 |
| Zhang (a) et al. 2017 | * | N/A | * | N/A | ** | * | * | * | 7/7 |
| Miao et al. 2017 | * | N/A | * | N/A | ** | * | * | * | 7/7 |
| Li et al.  2017 | * | N/A | * | N/A | ** | * | / | / | 5/7 |
| Rubin et al. 2017 | * | N/A | * | N/A | ** | * | * | * | 7/7 |
| Wang et al. 2017 | * | N/A | * | N/A | ** | * | * | * | 7/7 |
| Hinyard et al. 2017 | * | N/A | * | N/A | / | * | * | * | 5/7 |
| Zhang (b) et al. 2017 | * | N/A | * | N/A | ** | / | * | * | 6/7 |
| Wu et al. 2017 | / | N/A | * | N/A | ** | * | * | * | 6/7 |
| Ai et al. 2021 | / | N/A | * | N/A | ** | / | * | * | 5/7 |
| Alyabsi et al.2021 | * | N/A | * | N/A | ** | * | * | * | 7/7 |
| Ayaz et al. 2022 | * | N/A | * | N/A | ** | * | * | * | 7/7 |
| Cai et al. 2020 | * | N/A | * | N/A | ** | * | * | * | 7/7 |
| Ding et al. 2021 | / | N/A | * | N/A | ** | * | * | * | 6/7 |
| Liang et al. 2021 | * | N/A | * | N/A | ** | / | * | * | 6/7 |
| Wu et al. 2022 | * | N/A | * | N/A | ** | * | * | * | 7/7 |
| Xing et al. 2021 | / | N/A | * | N/A | ** | / | * | * | 5/7 |
| Khan et al. 2019 | * | N/A | * | N/A | ** | * | * | * | 7/7 |
| Wang et al. 2018 | * | N/A | * | N/A | ** | * | * | * | 7/7 |

*Note. One * represents one point. N/A – not applicable.*
